# A cutaneous Anthrax outbreak in Koraput District of Odisha-India 2015

**DOI:** 10.1186/s12889-019-6787-0

**Published:** 2019-05-10

**Authors:** Priyakanta Nayak, Samir V. Sodha, Kayla F. Laserson, Arun K. Padhi, Basanta K. Swain, Shaikh S. Hossain, Aakash Shrivastava, Pradeep Khasnobis, Srinivas R. Venkatesh, Bikash Patnaik, Kailash C. Dash

**Affiliations:** 1 National Centre for Disease Control, 22 Shamnath Marg, Civil Lines, New Delhi, India; 2 Directorate of Health Services, Bhubaneswar, Odisha India; 3 United States Centers for Disease Control and Prevention, Delhi, India; 4 Division of Global Health Protection, Centers for Global Health, Centers for Disease Control and Prevention, Atlanta, USA

**Keywords:** Anthrax, Koraput, India, Global health security

## Abstract

**Background:**

Cutaneous anthrax in humans is associated with exposure to infected animals or animal products and has a case fatality rate of up to 20% if untreated. During May to June 2015, an outbreak of cutaneous anthrax was reported in Koraput district of Odisha, India, an area endemic for anthrax. We investigated the outbreak to identify risk factors and recommend control measures.

**Method:**

We defined a cutaneous anthrax case as skin lesions (e.g.*,* papule, vesicle or eschar) in a person residing in Koraput district with illness onset between February 1 and July 15, 2015. We established active surveillance through a house to house survey to ascertain additional cases and conducted a 1:2 unmatched case control study to identify modifiable risk factors. In case control study, we included cases with illness onset between May 1 and July 15, 2015. We defined controls as neighbours of case without skin lesions since last 3 months. Ulcer exudates and rolled over swabs from wounds were processed in Gram stain in the Koraput district headquarter hospital laboratory.

**Result:**

We identified 81 cases (89% male; median age 38 years [range 5–75 years]) including 3 deaths (case fatality rate = 4%). Among 37 cases and 74 controls, illness was significantly associated with eating meat of ill cattle (OR: 14.5, 95% CI: 1.4–85.7) and with close handling of carcasses of ill animals such as burying, skinning, or chopping (OR: 342, 95% CI: 40.5–1901.8). Among 20 wound specimens collected, seven showed spore-forming, gram positive bacilli, with bamboo stick appearance suggestive of *Bacillus anthracis*.

**Conclusion:**

Our investigation revealed significant associations between eating and handling of ill animals and presence of anthrax-like organisms in lesions. We immediately initiated livestock vaccination in the area, educated the community on safe handling practices and recommended continued regular anthrax animal vaccinations to prevent future outbreaks.

## Background

Cutaneous anthrax, a zoonotic disease caused by *Bacillus anthracis*, can have a case fatality rate up to 20% if untreated; case fatality reduces to < 1% if treated [1]. *Bacillus anthracis* is found naturally in soil in a spore form and commonly infects domestic and wild animals around the world. Anthrax in animals’ spreads to humans after close proximity and interactions. Human anthrax occurs regularly in countries where environmental conditions are conducive for spore survival in soil and widespread vaccination of animals is not practiced.

In most patients (95%), the disease manifests as cutaneous anthrax, whereas the remaining 5% of cases present with inhalational or gastrointestinal syndromes. Cutaneous anthrax generally develops after direct contact with infected animals or animal products [2]. In South Asia, anthrax is highly endemic particularly in India and Bangladesh where frequent outbreaks and cases are reported among animals and humans despite limited laboratory and epidemiologic capacity. India has the world’s largest livestock population; animal anthrax is reported from several regions of the country [1].

Human anthrax in India is a notifiable disease. It commonly affects communities living near forest areas and outbreaks are predominantly reported from eastern states such as Odisha. Koraput is a tribal district in Odisha and is endemic for animal anthrax with poor vaccination among the livestock reported. Human outbreaks have been reported every year in Koraput since 2002, but limited coordination between the animal and human health sectors have led to incomplete laboratory confirmation and limitations in the detection, control and prevention of these outbreaks. Rapidly detecting and controlling public health emergencies, such as anthrax outbreaks, at their source is an important goal of the World Health Organization’s International Health Regulation; meeting the IHR obligations enhances global health security.

On March 25, 2015, two human cases of skin ulcers were reported to Odisha’s Integrated Disease Surveillance Programme (IDSP) as possible anthrax. We investigated to understand the epidemiology, identify risk factors associated with illness, and to propose recommendations for prevention and control.

## Methods

### Historical review of outbreaks

The India EIS officer investigating the outbreak reviewed available historical data from the Koraput district IDSP to identify demographic, clinical, and seasonal characteristics of suspected cutaneous anthrax outbreaks reported in this area from 2010 to 2014.

### Case finding

We defined a *suspect case* as a painless skin lesion such as a vesicle, eschar, or ulcer in a resident of Koraput district with illness onset between February 1 and July 15, 2015, and a *probable case* as a suspect case with microscopic evidence from a skin lesion suggesting *Bacillus anthracis*.

We defined a *high-risk village* as any village in Koraput district with reported cases of suspected anthrax (human or animal) in the last 5 years and an *affected village* as a village in Koraput district with reported human cases between February 1 and July 15, 2015.

We conducted enhanced passive surveillance through review of medical records of all patients from the district hospital in Koraput between February 1 and July 15, 2015 and active surveillance by implementing a house-to-house survey among high risk and affected villages in Koraput district between June 1 and July 15, 2015.

### Case control study

As this was a public health response conducted as part of the Indian EIS Programme, local permission was sought and given. We conducted a 1:2 unmatched case control study to identify risk factors for anthrax. All suspect cases between May 1 and July 15, 2015 were enrolled. We selected controls by finding the nearest neighbour of a case without any skin lesions from May 1 to July 15, 2015.

Data were collected using a standardized questionnaire to capture demographic details and suspected risk factors like handling an animal that died of illness. Frequencies and odds ratios were calculated using Epi Info 7.1.4.

### Human laboratory investigation

We collected wound swabs from cutaneous lesions of cases and processed them for Gram stain at the district headquarter hospital laboratory in Koraput. The Koraput district headquarter laboratory does not have capacity to do bacterial culture.

### Animal and environmental investigation

We reviewed records from the district animal health department regarding the district livestock population and deaths including those of cows, bulls, buffaloes, and goats. The records on livestock vaccination campaigns conducted by the district were reviewed.

We also created and evaluated spot maps of animal and human cases to see if there was clustering of human anthrax cases around locations of animal deaths.

The animal health department collected 250 random blood samples for culture from livestock among outbreak households which included cows, bulls and buffaloes in Koraput district during the months of April and May.

Soil samples were also collected for culture from two dead animal sites in the village. Specimens were processed for culture at the Animal Disease Research Institute in Cuttack district of Odisha which is the state animal reference laboratory.

## Results

### Historical review of outbreaks

**Fig. 1:**
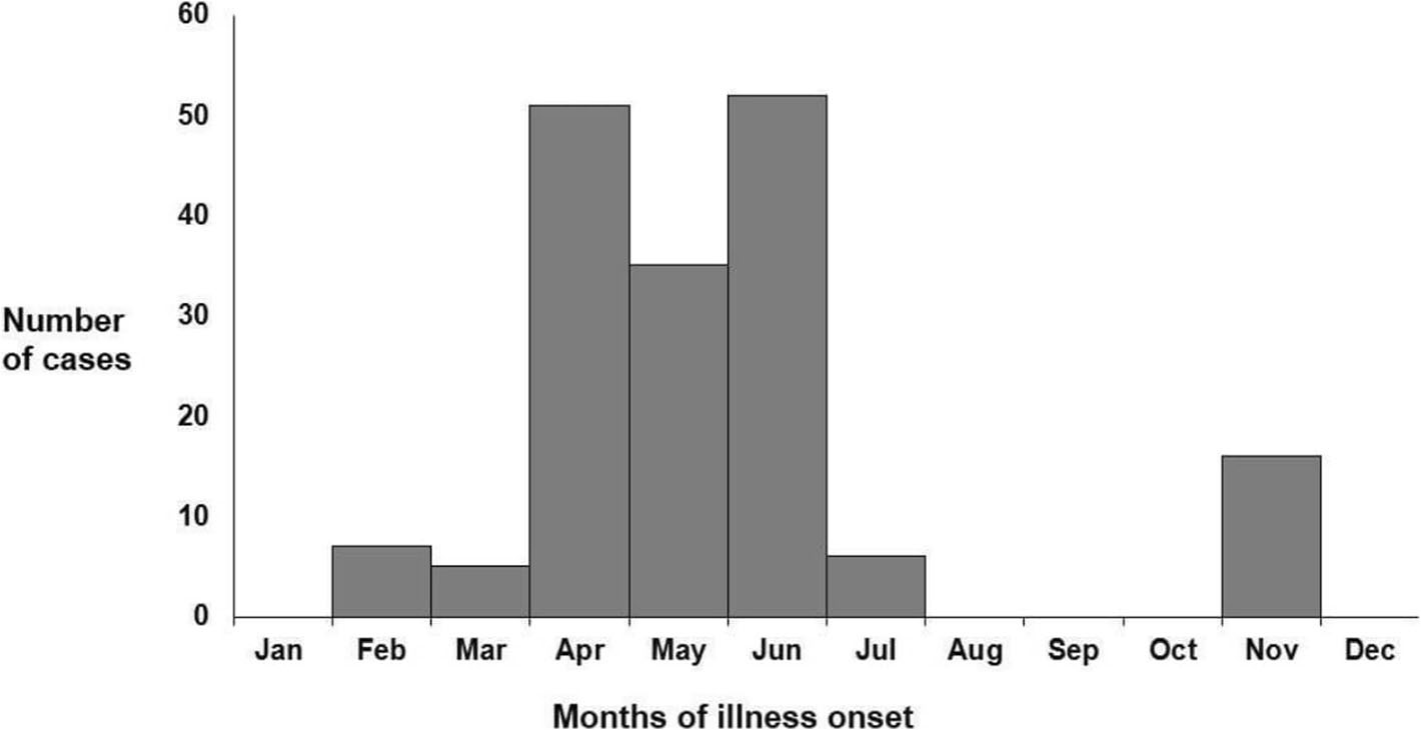
Monthly distribution of anthrax cases, Koraput, Odisha, India, 2010–2014 (*n* = 172)

From 2010 to 2014, we identified nine outbreaks of anthrax in Koraput with 325 suspected anthrax cases and 5 deaths (case fatality rate = 2%). Among the 325 cases, 82% were males and the median age was 36 years (range 4–78 years). The median time between appearance of cutaneous lesion and treatment initiation was 2 weeks. Anthrax cases in Koraput from 2010 to 2014 showed a seasonal trend (Fig. 1) with the majority of cases (80%) reported from April to June.

### Descriptive epidemiology of 2015 outbreak

**Fig. 2:**
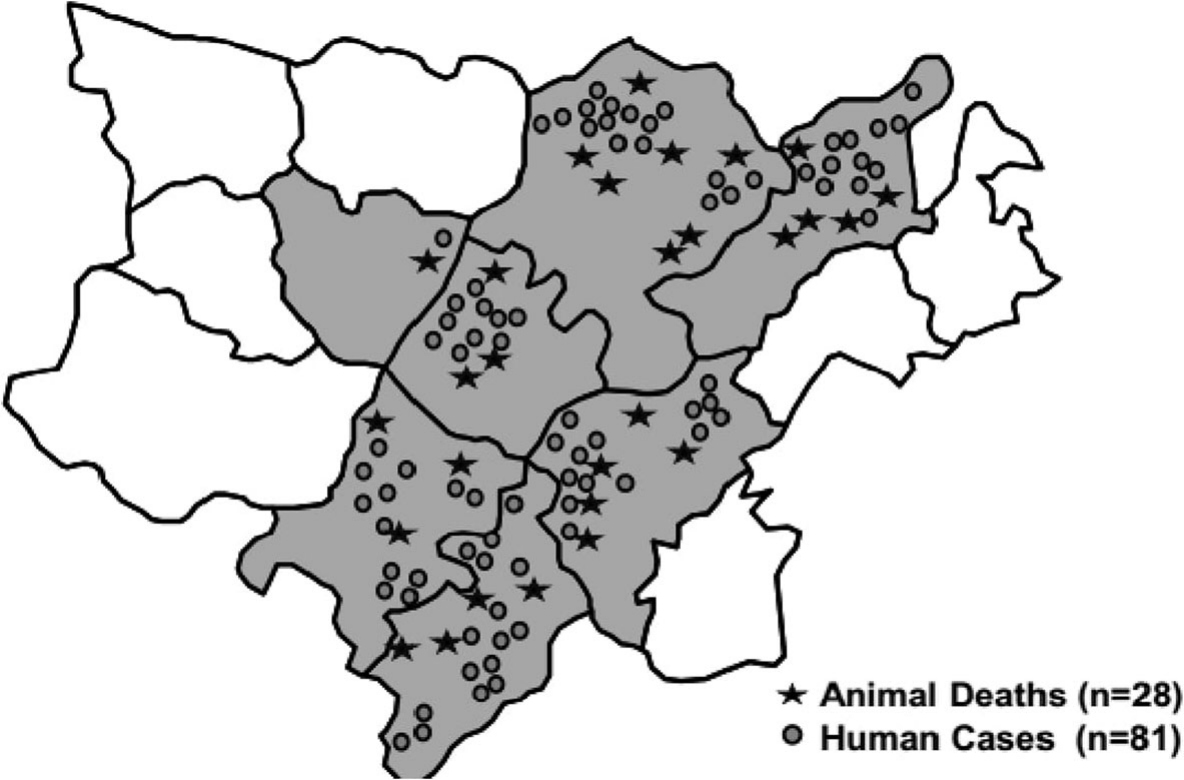
Animal deaths and human anthrax cases, Koraput, Odisha, India-2015

In the 7 affected sub-districts of Koraput district, we identified 81 cases (74 suspected and 7 probable cases) in 20 villages (Fig. 2). All were from the local tribal community with an average monthly income of INR 1800 (30 US dollars). Among the cases, 89% were males, and the median age was 38 years (range 5–72 years); there were 3 deaths (case fatality rate = 4%). All cases had cutaneous lesions such as eschars; 16% had malaise and 10% fever. No cases reported shortness of breath or bloody diarrhoea.

**Fig. 3:**
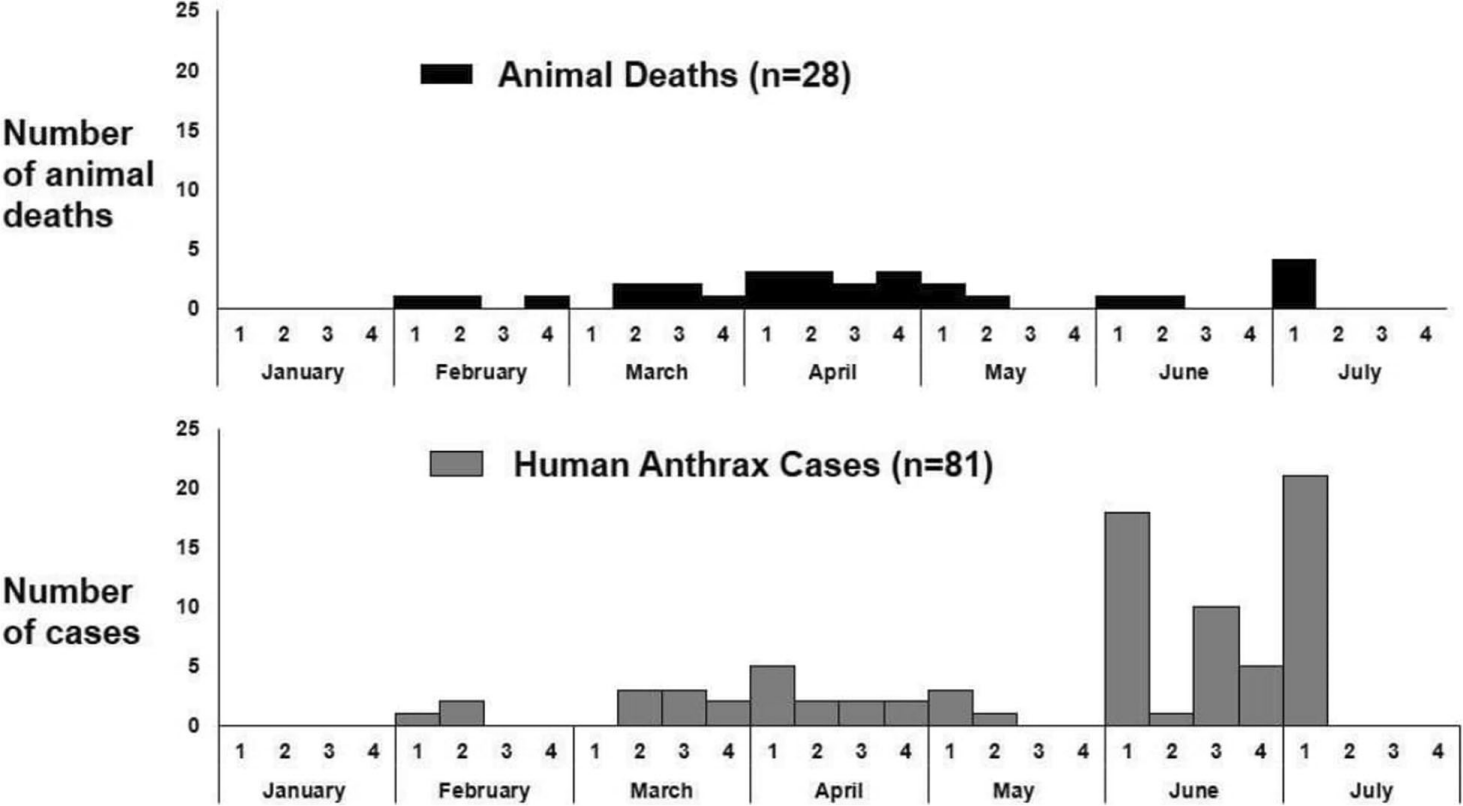
Human anthrax cases by week of illness onset and animal deaths by week of occurrence, Koraput, Odisha, India-2015

The onset of the outbreak occurred the 1st week of February 2015, continuing till the 2nd week of July with 2 peaks in the months of June and July respectively. There were no cases reported after the first week of July (Fig. 3).

### Case control study

Among 37 cases and 74 controls, illness was significantly associated with closely handling carcasses of ill cattle such as burying, skinning, or chopping (OR: 342, 95% CI: 40.5–1901.8) and with eating meat of ill cattle (OR: 14.5, 95% CI: 1.4–85.7) and (Table 1). There was no association of illness with domestic cattle contact, cultivation, visiting forest and washing meat of ill cattle.

**Table 1:** Risk factors associated with the anthrax cases in Koraput, Jan –June 2015 (*n* = 81)

Exposure	Case (*n* = 37)	Control (*n* = 74)	Odds Ratio	95% CI
Eating meat of ill cattle	100%	73%	14.5	(1.4–85.7)
Domestic animal contact	97%	93%	2.6	(0.3–23.1)
Handling carcass of ill cattle^a^	84%	0	342.1	(40.5–1901.8)
Visiting forest	81%	69%	1.9	(0.74–5.04)
Cultivation	78%	76%	1.1	(0.4–3.0)
Washing meat of ill cattle	5%	8%	0.6	(0.1–3.4)

^a^involves burying, skinning, or chopping

### Laboratory investigation (human)

Among 20 collected wound samples, 35% (7/20) showed large, gram positive bacilli consistent with *Bacillus anthracis***.**

### Environmental / animal investigation

Among 250 livestock blood specimens, 3 (1%) were culture positive for *Bacillus anthracis.* Soil samples collected from two dead cattle handling sites showed no growth. We found 28 cattle deaths occurring at the same time of human anthrax cases (Fig. 2)**.** There is no routine vaccination program for livestock in Koraput district. During the outbreak response, 6900 cattle within a 5 km radius of the 20 affected villages were vaccinated by anthrax spore vaccine. No other livestock were vaccinated. The vaccination coverage of livestock before the outbreak was < 1% (1833/305,497) and then 2% (6900/305,497) after the vaccination campaign.

## Discussion

We report on a cutaneous anthrax outbreak mostly among males in a tribal community of Koraput district, Odisha. This community has a history of recurrent anthrax outbreaks that occur seasonally, from April to June. Our investigation demonstrated clustering of human cases in areas of animal deaths with human illness strongly associated with eating and handling the carcasses of ill cattle Low vaccination coverage of livestock and inadequate carcass disposal practice in affected sub-districts likely contributed to the outbreak and to the ongoing risk in the community.

Anthrax continues to be enzootic in under-developed areas of the world lacking adequate preventive measures [3]. Rapid diagnosis, isolation, treatment with antimicrobials and other adjuvant therapies among human anthrax cases and measures against transmission are essential to minimize disease progression and to help control outbreaks quickly and effectively. With 1% prevalence of culture positivity among outbreak household cattle, Koraput district has endemic livestock anthrax and inadequate vaccination coverage; these factors likely contribute to continuous transmission of anthrax in this region [4].

Socio-cultural practices such as slaughtering of sick animals, eating or handling meat from infected animals, and dumping of dead carcasses in the open have contributed to anthrax transmission in outbreaks reported from Africa and Southeast Asian countries [4]. Scavenging carcass meat for consumption is culturally acceptable to some of the local tribes in Koraput district and is associated with anthrax transmission. Low socioeconomic status and poor education of the tribal community combined with poor public health infrastructure creates a synergy of risk factors that are conducive for zoonotic transmission of anthrax to the human population [5]. Increasing the tribal community’s awareness about risk factors for illness may help avert future outbreaks. As part of the response to this outbreak, health education camps were organized to sensitize the community on behavioral change for anthrax prevention. All the villagers were educated through simple health messages from the community health workers such as “not to handle the sick animal without protection,” “safe disposal of dead animal with disinfection,” “not to consume raw meat,” “cook it well before eating,” etc.

As part of the outbreak investigation, a daily reporting system with active surveillance was established in the vulnerable villages and contact tracing was done. In previous outbreaks, delayed reporting of ill persons and poor community awareness about the illness were important risk factors for continued transmission of infection. Furthermore, unreported livestock deaths and seasonal variation of anthrax transmission in the district may also contribute to the persistence of outbreaks [6].

Strengthening district level laboratory capacity for anthrax is crucial for early identification of an outbreak. Lack of anthrax culture capacity or other diagnostic methods in the district laboratory limits detection of anthrax in animal, human and environmental samples. There is need to strengthen the laboratory surveillance system at the district level both for animal and human health, and the building of a more reliable sample transportation and communication system. The lag-phase between reporting of the outbreak and the specimen sample collection often leads to delayed diagnosis and confirmation of the outbreak.

After outbreak detection, enhanced skills in field epidemiology are necessary for rapid response and thorough investigation. India is actively working toward improving epidemiologic capacity quantitatively and qualitatively. In 2012, the India Epidemic Intelligence Service (EIS) programme was launched by India’s National Centre for Disease Control, Delhi in collaboration with the United States Centers for Disease Control and Prevention. This 2-year intensive training in field epidemiology aims to increase the number of public health professionals with specialized skills to investigate outbreaks with analytical epidemiology. This outbreak investigation was led by an EIS officer and demonstrates the benefit of applying these skills in India to better understand active public health problems and guide evidence-based interventions.

The epidemiology of anthrax involves human, animal, and environmental health [7]. The One Health approach integrates and synergizes these multiple disciplines toward disease control and prevention strategies of zoonotic diseases such as anthrax [8]. We used this outbreak investigation as an opportunity to strengthen the inter-sectoral coordination between the human and animal health departments in the district. After the outbreak was identified among human cases, the animal health department was engaged to trace the tracks and hides of infected animals, treat infected animals, and immediately destroy dead carcasses. While surveillance for human cases was ongoing, the dispatching and transaction of livestock from the area was stopped. Such coordination between animal and human health departments, as is promoted under the Global Health Security Agenda, will facilitate early case detection, control, and prevention of zoonotic disease outbreaks in the future.

Seasonality of anthrax outbreak is a complex phenomenon. Several studies have hypothesized that seasonality, climate conditions and/or human activities are associated with anthrax outbreaks, but there is minimal evidence about their association [9]. However, outbreak patterns vary from region to region based on the diverse predisposing environmental conditions existing in the area. The majority of the anthrax outbreaks in India are reported between the months from September to January (post-monsoon). In contrast, our investigation of historical anthrax cases in Koraput showed a predominance of cases occurring from April to June (pre-monsoon)*.*

Our investigation is subject to certain limitations. First, there was recall bias in the case control study as cases were interviewed up to 4 weeks after illness of onset. However, despite this, we were still able to find strong statistical associations with illness. The rapid increase and decrease of human anthrax cases detected may have been due to a surveillance artifact from increased and decreased surveillance activities. Also, the limited laboratory capacity for anthrax culture in Koraput district in both human and animal health side limited our ability to confirm the etiology. Finally, the village animal health records had limited data available regarding migratory patterns and vaccination history though we were still able to show geographical clustering.

## Conclusion

Global health security relies on all countries having the capacity to rapidly detect and control public health emergencies, such as an outbreak of anthrax, at its source. Anthrax is a re-emerging disease of public health importance in India. Livestock vaccination, surveillance for early case detection, and education among the community regarding animal to human transmission and carcass disposal is crucial for prevention, detection, and control of outbreaks. The vaccination strategy of animals should expand to larger geographic areas with more comprehensive goals to vaccinate more livestock and variety of livestock.

## Abbreviations

CDC: Centers for Disease Control and Prevention
EIS: Epidemic Intelligence Service
IDSP: Integrated Disease Surveillance Program
IHR: International Health Regulation
WHO: World Health Organization
